# Synergy-Based Bilateral Port: A Universal Control Module for Tele-Manipulation Frameworks Using Asymmetric Master–Slave Systems

**DOI:** 10.3389/fbioe.2017.00019

**Published:** 2017-04-03

**Authors:** Anais Brygo, Ioannis Sarakoglou, Giorgio Grioli, Nikos Tsagarakis

**Affiliations:** ^1^Department of Advanced Robotics (ADVR), Istituto Italiano di Tecnologia (IIT), Genova, Italy; ^2^Interdepartmental Research Center “E. Piaggio”, Faculty of Engineering, University of Pisa, Pisa, Italy

**Keywords:** teleoperation, tele-manipulation, synergy, asymmetric systems, exoskeleton, underactuation

## Abstract

Endowing tele-manipulation frameworks with the capability to accommodate a variety of robotic hands is key to achieving high performances through permitting to flexibly interchange the end-effector according to the task considered. This requires the development of control policies that not only cope with asymmetric master–slave systems but also whose high-level components are designed in a unified space in abstraction from the devices specifics. To address this dual challenge, a novel synergy port is developed that resolves the kinematic, sensing, and actuation asymmetries of the considered system through generating motion and force feedback references in the hardware-independent hand postural synergy space. It builds upon the concept of the Cartesian-based synergy matrix, which is introduced as a tool mapping the fingertips Cartesian space to the directions oriented along the grasp principal components. To assess the effectiveness of the proposed approach, the synergy port has been integrated into the control system of a highly asymmetric tele-manipulation framework, in which the 3-finger hand exoskeleton HEXOTRAC is used as a master device to control the SoftHand, a robotic hand whose transmission system relies on a single motor to drive all joints along a soft synergistic path. The platform is further enriched with the vision-based motion capture system Optitrack to monitor the 6D trajectory of the user’s wrist, which is used to control the robotic arm on which the SoftHand is mounted. Experiments have been conducted with the humanoid robot COMAN and the KUKA LWR robotic manipulator. Results indicate that this bilateral interface is highly intuitive and allows users with no prior experience to reach, grasp, and transport a variety of objects exhibiting very different shapes and impedances. In addition, the hardware and control solutions proved capable of accommodating users with different hand kinematics. Finally, the proposed control framework offers a universal, flexible, and intuitive interface allowing for the performance of effective tele-manipulations.

## 1 Introduction

Performing a task using a teleoperated robot avatar makes possible to safely handle hazardous material as well as to take advantage of the machines’ superior sensing and actuation capabilities, enabling the execution of tasks that require high precision and large wrenches. Furthermore, the introduction of a digital control layer between the operator’s commands and the actuator’s reference output allows to resort to signal processing techniques such as scaling or filtering in order to rectify the user’s input. Additionally, preoperative automated task supervision features can be implemented to improve safety with, for instance, the introduction of motion constraints and safeguards to contain the risks stemming from accidental commands or external disturbances. As such, teleoperation not only enables to project the skills of an operator into a remote environment but it also permits to extend and enhance the human’s manipulation capabilities with higher power, higher motion precision, and with greater safety. Finally, this powerful tool allows merging the strengths of men and machines so as to achieve enhanced performances. However, to take advantage of the potential benefits offered by tele-manipulation, a number of considerations need to be addressed both at the hardware and software levels.

### 1.1 Coping with the Master–Slave Asymmetry

Of paramount importance in the realization of an intuitive tele-manipulation platform is the design of a bilateral control module capable of efficiently coping with diversely asymmetric master–slave systems.

To better comprehend this matter, one should bear in mind that the inherent capabilities of a teleoperator are conditioned by those of its master and slave devices, whose choice is guided by distinct functional requirements. On the one hand, the slave robot must incorporate an end-effector that is suitable for the task, i.e., that achieves the most adequate versatility–robustness trade-off to effectively manipulate its environment. Depending on the manipulation scenario considered, the end-effector may be chosen to be anything from a highly anthropomorphic dexterous hand to a single DoF industrial gripper. On the other hand, the master device should allow for unconstrained motions in the entire user’s hand workspace and provide an accurate posture tracking as well as a rich haptic guidance while maintaining the degree of portability required by the application.

These criteria often lead to the selection of highly asymmetric systems at the kinematics, sensing, and actuation levels. Such asymmetry, if not carefully handled, might greatly decrease the intuitiveness of the framework and consequently jeopardize its effectiveness. The core strategy to make the operator unaware of the system’s asymmetry and allow him/her to command manipulation actions as if directly performing the manual task is to capture his/her intent during natural motions and map this input signal from the master sensing space to the slave’s actuator space so as to generate motion references translating the desired actions. Conversely, an adequate algorithm is needed to synthesize, from the data sensed at the slave side, a force feedback reference for the master’s actuators in order to display an effective haptic guidance capable of enabling the operator to immerse in the task. Those policies play a major role in isolating the user from the complexity of the asymmetric system so as to create an efficient human–machine interface, where the natural coupling with the slave makes possible the execution of complex manipulation tasks while maintaining low the cognitive load of the operator.

Finally, tele-manipulators belong to the larger class of Human-In-the-Loop (HIL) systems. As such, their overall performance not only depends on the capabilities of the master and slave devices in isolation but also on how well these two entities are interconnected. It is therefore important to develop bilateral control strategies that effectively cope with the master–slave asymmetries.

### 1.2 On the Need of Unified Control Frameworks

Besides handling the master–slave asymmetry, it is desirable to design flexible and universal control frameworks.

Indeed, in an attempt to achieve the versatility required to accommodate the wide range of shapes and sizes of the objects populating our workspaces, artificial hands exhibiting increasingly sophisticated designs have been developed during the last decade. However, the effective use of such highly articulated devices has been hindered by their inherent control complexity. A promising approach to tackle this difficulty lies in the development of unified frameworks promoting the synthesis of hardware-independent algorithms that can be indifferently used to control a variety of end-effectors. This can be achieved through adopting a two-layer architecture, where the low-level layer is used to encapsulate the kinematics, sensing, and actuation specifics of the device, while the high-level layer focuses on the resolution of the manipulation problem. This latter entity, which may be seen as equivalent to the concept of middleware in software engineering, can be based on universal, task-oriented control policies designed in complete abstraction from the hardware to be used. Provided the use of a generic interlayer interface, such platform permits to flexibly interchange not only the robotic hand used but also the control layer, making possible to easily implement a diversity of manipulation algorithms proposed in the literature, from autonomous or semi-autonomous grasp planners, as the one presented in Ciocarlie et al. (2007), to tele-manipulation strategies designed in the object domain, as proposed in Gioioso et al. (2013), or any other mapping that best suits the application considered.

The implementation of interfaces as generic as possible would benefit from the selection of a common expression space. As such, the concept of postural synergies has been proposed as the alphabet of the universal language for such unified control frameworks. This bio-inspired approach rests on neuroscience studies that have shown the existence of consistent spatiotemporal coordination patterns of the human hand joints during the execution of a variety of manipulation tasks (Santello et al., 2016). Among other possible projection spaces, the synergistic one appears very attractive for two main reasons. First, it seems sensible to draw inspiration from the CNS’s^1^ strategy to control the human hand that remains to date and by far the most complex and versatile manipulation tool existing. Second, considering the postural synergies as motion primitives that can be combined to design manipulation strategies can significantly decrease the control complexity. Indeed, analysis has revealed that a few variables, corresponding to the coordinates along the synergistic directions, can describe most of the variance of the hand posture during grasping motions (Santello et al., 1998).

Hence, deriving control algorithms in the synergy space can substantially reduce the dimensionality of the manipulation problem and therefore the associated computational cost.

To complete such architecture, low-level modules are required to translate the references described in synergy space into equivalent patterns of coordinated motion of the artificial hands’ joints. While this mapping is quite straightforward when considering anthropomorphic robotic hands, the derivation of optimal posture subspaces for end-effectors with kinematics considerably different to those of the human hand remains an open research question. A first contribution in this direction has been proposed in Ciocarlie and Allen (2010), where the authors empirically define a set of basis vectors, referred to as eigengrasps, which describe a low-dimensional posture subspace for 5 artificial hands. This very promising work demonstrates the applicability of such approach and paves the way to the generalization of robotic hands control within a synergy-based unified framework.

### 1.3 The Approach Proposed

This present work introduces a novel bilateral tele-manipulation control strategy that resolves the asymmetry of the master–slave system and generates motion and force feedback references in the hardware-independent hand postural synergy space.

In this tele-manipulation platform, the 3-finger hand exoskeleton HEXOTRAC (Sarakoglou et al., 2016) is used at the master station of the proposed platform. Its sensory and actuation systems permit to monitor the position and orientation of the operator’s fingertips and to display kinesthetic feedback reflecting some mechanical properties of the remote environment. This exoskeleton is used to control the Pisa/IIT SoftHand (SH), an anthropomorphic robotic hand, the as slave device. This device implements the concept of soft and adaptive synergies mechanically, such that its 19 joints are driven by a single motor along the first synergy in free space, while its shape adaptation capabilities stemming from underactuation let it conform to the shape of objects during grasping. With a single actuator and a sensory system limited to a position encoder and a current sensor, the SoftHand’s minimalistic design promotes robustness but challenges traditional teleoperation strategies. Indeed, the absence of joint encoders and individual actuators prevents the control of the slave’s joints or fingertips trajectories as a straightforward mapping of the operator’s motions. Similarly, the absence of per-digit haptic sensors calls for a novel force feedback control strategy.

**Figure 1:**
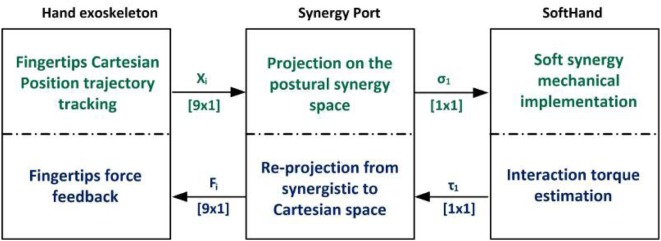
**Introduction of a synergy port as a mapping tool for bilateral tele-manipulation**.

To tackle this challenge and achieve an intuitive framework capable of efficiently handling the large asymmetry between the master and the slave devices, the concept of Cartesian-based hand synergies is introduced as a set of independent vectors oriented along the grasp principal components and described in the fingertip Cartesian space. This base is used to develop a novel bilateral synergy port, schematized on Figure 1, which is proposed as a mapping tool for performing bilateral tele-manipulations.

In the proposed approach, the operator’s hand posture is projected on this space to extract the corresponding coordinate along the first synergy, which is then used as the SoftHand’s motor position reference. This algorithm elegantly scales the human hand’s many degrees of freedom to the slave’s single Degree Of Actuation (DOA) by extracting from natural motions the relevant information to be used as input for operating the slave—here the desired degree of hand closure. Conversely, the proposed synergy port is used to generate force feedback references for the exoskeleton to display perceptually meaningful haptic stimulations that characterize the remote interaction. The 1-dimensional grasping force applied by the SoftHand’s actuator on its environment along the first synergy is estimated using a torque observer. It is then re-projected on the Cartesian-based synergy space in order to synthesize a 9-dimensional force reference in the user’s fingertip Cartesian space. These finger-individualized forces, which reflect the grasping effort developed by the SoftHand during its interactions with the objects manipulated, are finally projected on the exoskeleton’s joint space in order to generate the reference for its actuated DOFs. To complete the tele-manipulation interface, the framework is subsequently enriched with the vision-based motion capture system Optitrack, which is used to monitor the position and orientation of the user’s wrist. This input is used to generate in real time the 6D position reference trajectory for the wrist of the robot arm on which the SoftHand is mounted. This control scheme has been implemented to control the IIT humanoid robot COMAN as well as the KUKA LWR manipulator.

This work builds upon the proof-of-concept study presented in Brygo et al. (2016), which demonstrated the effectiveness of the proposed synergy-based interface in allowing a single trained user to perform the desired tele-manipulation. This preliminary work is developed and extended here in order to achieve a universal platform. In particular, the theoretical and practical tools needed to endow the framework with the capability to accommodate very different users’ hand sizes are implemented, and the usability of the interface by novice, untrained operators is experimentally assessed.

The rest of this paper is organized as follows. In Section 2, a description of the hand exoskeleton and the SoftHand is presented along with the principal characteristics that motivated the selection of these devices. A brief review of the strategies proposed in the literature to control robotic hands using asymmetric teleoperators is then presented, and the need for a novel mapping strategy suiting the proposed system is explained. Section 3 addresses the generation of the SoftHand’s motor position reference as a mapping of the user’s hand posture. In particular, the concept of Cartesian-based hand synergy is introduced together with the user study that has been conducted to analyze the possibility to describe the human hand posture during grasping motions using a low-dimension set of variables constructed as a linear combination of the fingertip Cartesian trajectories. Subsequently, the theoretical tools used to build the synergy-based teleoperation port are derived. The effectiveness of this approach to synthesize the artificial hand motion reference realizing the operator’s intentions is then experimentally demonstrated. Section 4 describes how the force feedback references are generated through inverse projection on the synergy space, and a finger-individualized force scaling procedure is proposed to homogenize the force feedback amplitudes across users. The closed-loop performance of the system is then experimentally characterized. Section 5 discusses the possibility of relying on a simplified calibration procedure and analyzes how the use of a user-independent Cartesian-based synergy matrix affects the position and force feedback references. Section 6 presents the experiments conducted with both robotic slaves in order to evaluate the effectiveness of the proposed framework. Finally, conclusions of this work are drawn in Section 7.

## 2 A Highly Asymmetric Master–Slave System

### 2.1 A Hand Exoskeleton at the Master Station

A fundamental aspect to consider when designing tele-manipulation interfaces concerns the choice of the master device. One option to monitor the user’s hand posture consists in tracking the fingers’ joints’ angular excursion. Vision-based motion capture systems can be used to this end. While flexibly adjusting to different hand sizes, their main drawback regards the need to handle occlusions, an issue made particularly salient by the large number of markers in a small workspace. Furthermore, the installation of a large number of markers on the skin is a time-consuming procedure. Alternatively, data gloves such as the VPL data glove (Hong and Tan, 1989) or the CyberGlove from Immersion Corporation (Peer et al., 2008) represent a popular solution that does not suffer from this limitation. However, reservations have been expressed about their tracking accuracy. Indeed, not only it can be affected by physical factors such as sweat and temperature but it is also largely dependent on the fit of the glove. Accommodating diverse hand sizes is a critical requirement when aiming at designing a universal interface, as it is the case of this work. Calibration procedures have been developed to account for the different hand kinematics (Griffin et al., 2000), but besides consisting in tedious processes that can be time consuming, their results are often not ideal (Dipietro et al., 2008). Finally, additional solutions need to be added in order to enhance the master station with force feedback capabilities, which is desirable to enable remote haptic explorations and is believed to assist the operator in regulating the manipulation forces.

**Figure 2:**
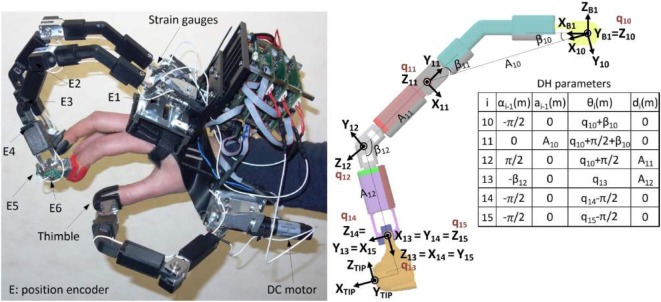
**Left: hand exoskeleton HEXOTRAC; right: kinematics of the exoskeleton’s index finger linkage**.

These considerations motivated our choice to use the hand exoskeleton HEXOTRAC (Sarakoglou et al., 2016), pictured in Figure 2, at the master station of our tele-manipulation framework. It consists of a three 6-DOF linkages mounted on a common base, which is attached to the dorsal side of the hand. The other end of the linkages is fastened to the user’s thumb, index, and middle fingertips through interchangeable 3D-printed thimbles. This attachment system not only allows for fast donning and removal of the device but it also permits to suit a large range of hand sizes. The overall kinematics design is such that the reachable workspace of the linkages endpoint completely covers the human hand workspace. This makes possible the execution of any type of motion and permits in particular the rotation of the fingertips, which provides the freedom to perform natural manipulations and is the key to accommodating a very large range of hand sizes without any mechanical adjustment. High resolution magnetic encoders monitor the position of all the linkages’ joints, permitting the 6-DoF tracking of the operator’s fingertips.

The hand exoskeleton is fitted with three low-gear DC motors whose output torques are transmitted to the first joint of each finger linkage through a pair of anti-backlash bevel gears. Pairs of bilaterally bonded strain gages provide measurement of the torque applied at the proximal joints. This measurement is used as feedback signal by a PD controller that tracks the reference torque trajectory. $\tau_{\mathrm{int}\;\mathrm{des}}\in R^{3n_{a}}$ is the joint torque vector corresponding to the desired fingertip interaction force projected on the actuated joint space. Since the strain gages reading $\tau_{\mathrm{str}\;g}\in R^{3n_{a}}$ encompasses both the interaction torque and the gravity torque, a gravity compensator is introduced to compute $\tau_{g}\in R^{3n_{a}}$, the model-estimated gravity joint torque projected on the actuated joint space. Note that *n_a_* is the exoskeleton finger-actuated joint space dimension.

Finally, this hand exoskeleton is used as master system of the teleoperation system. The following section describes the robotic hand used as slave device.

### 2.2 The SoftHand at the Slave Station

The choice of the slave device is of paramount importance, since its intrinsic characteristics greatly influence the capabilities of the tele-manipulator as well as its intuitiveness. After introducing in Sect. 2.2.1 the efforts of the robotic community toward the development of robotic hands equipped with the technical capability to skillfully manipulate the many different objects populating our workspaces, the SoftHand’s characteristics that motivated its selection as the slave hand of the proposed tele-manipulation framework are presented in Sect. 2.2.2.

#### 2.2.1 Achieving Stable Grasp with Robotic Hands, a Long-lasting Challenge

Versatile robotic manipulation in unstructured environments is a challenging topic for the robotics community and a very active area of research during the last decade (Shimoga, 1996; Kemp et al., 2007). Of particular importance is the ability to manipulate objects with diverse geometries while precisely controlling the contact locations and force distribution in order to achieve force closure grasps, also referred to as stable grasps (Bicchi and Kumar, 2000; Okamura et al., 2000; Rosales et al., 2012; Prattichizzo et al., 2013). Considering the diverse shapes and sizes of the objects populating human workspaces, a considerable effort has been recently devoted to the development of highly dexterous artificial hands. The Utah/MIT hand (Jacobsen et al., 1984), the Anthrobot hand (Kyriakopoulos et al., 1997), the Shadow hand (Shadow Robot Company Ltd, 2017), or the DLR hands (Butterfass et al., 2001) are a few examples of such attempts to approach the fine manipulation capabilities of the human hands. However, their actual use has been largely hindered by their limited power capabilities and poor robustness, resulting from the implementation of many small and usually delicate actuators, as well as by their increased control complexity. Aware of these limitation, an innovative design has been proposed and implemented in the Pisa/IIT SoftHand (Catalano et al., 2012) used as slave device of the proposed tele-manipulation platform. The intrinsic properties of this hand, which conditioned the development of the control framework, are described in the following section.

#### 2.2.2 SoftHand, a Powerful Grasping Tool

The SoftHand has been selected because it elegantly combines versatility and robustness within a unique design that turns it into a powerful tool to grasp and manipulate objects of diverse shapes in unstructured environments.

**Figure 3:**
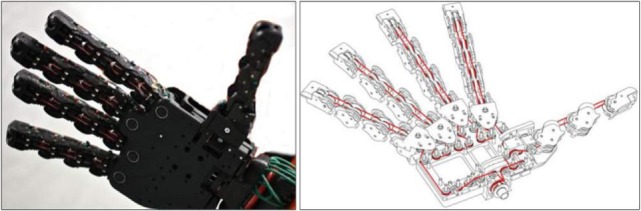
**Left: picture of the Softhand; right: routing of its tendon**.

Its transmission mechanism uses a system of pulleys and a tendon routed through all fingers to transmit the torque of a single motor to the 19 DOFs of the hand, as shown on Figure 3. Its differential system has been designed to drive all joints along the first synergy in free space motions, while during contact its shape-adaptive property deriving from underactuation lets it conform to the geometry of the object grasped. Such behavior is achieved through combining two main theoretical tools, such that the soft synergy concept is realized with a shape-adaptive underactuated mechanism. Indeed, while the neuroscience-based concept of the first postural synergy appears attractive since the manipulation of a single coordinate allows to describe a large percentage of the posture variance during natural grasps, a purely kinematic implementation of this synergy leads to an inconsistent grasp force distribution. The concept of soft synergies was proposed to tackle this limitation and provides a model generating suitable internal forces that permit to robustly hold an object (Bicchi et al., 2011).

In addition, an innovative design employing rolling contact articulations assembled with rubber ligaments allows the joints to disarticulate during impacts before passively returning to their initial configuration. These features endow the SoftHand with an exceptional robustness through ensuring a soft and safe behavior during unexpected collisions, making it suitable for manipulation tasks in unstructured environments.

Finally, this design embeds part of the control intelligence in the physical system itself. Indeed, while the actuation space is reduced to a single dimension, the SoftHand’s multiple DOFs move in a principled way and adapt to the environment manipulated, achieving high versatility at a reduced control complexity cost. However, these same characteristics challenge traditional teleoperation techniques, as discussed in the following section.

### 2.3 A Design That Challenges Traditional Teleoperation Strategies

With a single electric motor, one position encoder, and one current sensor, the SoftHand’s overall design aims to a low cost and robust device. However, such underactuation and minimum sensing represent a major challenge when considering the SoftHand as a slave device to teleoperate using a hand exoskeleton and prevents from relying on traditional teleoperation strategies. For a better understanding of the problematic, below are presented the different strategies that have been proposed in the literature to teleoperate robotic hand and cope with asymmetric master–slave systems.

There are three typical control strategies for teleoperating robotic hands: joint-to-joint, point-to-point, and pose mapping. Alternatively, an object-centered approach has been recently proposed. Following is a brief overview of these techniques.

Direct joint-to-joint mapping has been proposed as control strategy to teleoperate robotic hands (Wright and Stanisic, 1990; Bouzit, 1996; Yokokohji et al., 2003). This method, which appears suitable to drive anthropomorphic hands from recording the trajectory of the operator’s hand joints using motion tracking systems such as data gloves, presents some limitations. Indeed, the many DOFs of the human hand endow it with a remarkable dexterity that most robotic hands fail to achieve, thus creating a kinematic mismatch between the two sides. This in turn renders this approach of directly mapping the DoF of the slave hand to motions of the human hand joints unable to efficiently cope with this level of kinematic master–slave asymmetry.

As an alternative, point-to-point mapping has been proposed to control non-anthropomorphic hands or grippers. This approach consists in designing the reference trajectory of particular points of the robotic hand as a function of the Cartesian position trajectory of associated points of the master hand. Typically, the fingertips of the master hand are monitored and used to derive the desired position trajectory of the slave’s fingertips. In Rohling et al. (1993), the Utah/MIT Dextrous Hand is teleoperated using the Utah Dextrous Hand Master. In this work, the control algorithm aims at mapping the position and the orientation of master to the slave device. To account for the slight kinematic dissimilarities between the systems, a priority-based strategy is implemented such that position is considered as the prime goal while orientation comes as second objective. Another implementation reported in Hong and Tan (1989) proposes to teleoperated the Utah/MIT Dextrous hand using the VPL data glove. The position trajectories of the operator’s fingertips are computed from the measured joints position using the forward kinematics of the human hand model and then used to derive the reference trajectories of the slave fingertips. However, a direct mapping of the Cartesian trajectories of the fingertips might not be possible when considering larger asymmetries, such that alternative mapping might be needed. In Peer et al. (2008) is presented a method for teleoperating the three-fingered gripper BarrettHand using the CyberGlove as master tracking system. The control space of the slave’s hand is quite restricted since each finger counts two coupled DOFs actuated by a single motor; furthermore, two of the three fingers can rotate synchronously and symmetrically around the base joint. To generate references lying within the gripper’s reachable space, the workspace of the human fingers is scaled and then vertically projected.

The third approach, referred to as pose mapping, is based on the functional analysis of the human hand during natural actions. A collection of human poses or grasps are defined, and to each of them is associated an equivalent posture of the robotic hand. A transformation matrix is proposed in Pao and Speeter (1989) that maps human hand positions to corresponding positions in the slave space, and an interpolation algorithm is employed to shift from one pose to another. In Wojtara and Nonami (2004), a grasp recognition method relying on neural network is developed together with a custom mapping algorithm. The use of neural networks for grasp identification is also studied in Gorce and Rezzoug (2004). Alternatively, Ekvall and Kragic (2006) use Hidden Markov Models for the recognition of the human grasp, while a neural network is used to define the mapping between the operator’s and the slave’s hand configuration spaces. A grasp taxonomy is introduced in Liu and Zhang (2004), where a Gaussian Mixture Model based classifier is designed to recognize the type of grasp. Another strategy is used in Kang and Ikeuchi (1997) where a virtual finger mapping method is used after recognizing the human grasp in order to generate the slave’s reference. The main limitation of those strategies regards the poor robustness of the identification algorithms. As such, small changes in the operator’s hand posture can lead to switches between the reference pose in the target space, resulting in somewhat unpredictable motions that are especially undesirable during tele-manipulations.

A more recent approach has been proposed that focuses on manipulation tasks. The core concept consists in introducing a virtual object at the master station. The operator’s finger motions are translated into the rigid-body motion and deformation imparted on the virtual object. The slave hand is then controlled such that the object—either virtual or real—tracks the transformations of the virtual object. This paradigm aims at integrating the operator’s manipulation intent in the object domain. A 2D instance of such method is implemented in Griffin et al. (2000) and used to control a symmetric two-fingered planar robot. A non-linear mapping is developed to map the motion of the master virtual object to the motion of the slave virtual object in order to better utilize both station’s workspaces. This method is extended to 3D cases in Wang et al. (2005), where a 3-fingered robotic hand is teleoperated. A virtual circle is defined by the three considered fingertips of the operator. The master virtual circle is parameterized, and the parameter’s transformations are mapped to similar transformations of the virtual circle defined at the slave side. Finally, the robot’s finger motions are computed according to the desired fingertip position. An analogous approach is undertaken in Gioioso et al. (2013) where the smallest sphere enclosing the fingertips of the operator is used as virtual object. The user’s input is described by modeling the effects of the fingers’ displacement on this virtual sphere’s linear and angular velocities as well as it deformation. These transformations are scaled and tracked by a virtual sphere defined as the slave’s side. In Salvietti et al. (2013), the virtual object is used to both capture the user’s hand motion and to compute the force feedback reference, such that the wrench displayed to the user corresponds to the wrench applied by the robotic hand on the virtual object. Those object-based methods provide a universal interface that abstracts from the kinematics of the master and slave systems and therefore allows to cope with highly asymmetric systems.

However, none of these approaches can be used in the present work. Indeed, the extreme underactuation of the SoftHand and the absence of joint encoders in its fingers prohibit the design of the motion control reference in joint or Cartesian space as a traditional mapping of the operator’s motions. Furthermore, the absence of haptic sensors on the slave’s digits calls for a novel force feedback strategy in order to generate appropriate references for the hand exoskeleton’s actuators. Hence, the need to develop a novel bilateral teleoperation algorithm suitable for the system is considered in this work. This aspect is addressed in the following section.

## 3 Position Control: A Synergistic-Based Teleoperation Strategy

While the large number of DoFs affords the human hand with remarkable dexterity, the SoftHand’s underactuated kinematics, with its single actuator, limit control over the postures it can achieve. The purpose of the work presented in this section is to resolve this asymmetry through synthesizing a suitable algorithm that maps the native hand’s unconstrained motions into motions achievable by the robotic hand that best translate the user’s manipulation intent. To this end, we propose to capitalize on the insights recently gained with regard to the concept of postural synergies, which was revealed to be a key mechanism underlying the human hand motor control and which inspired the design of the SoftHand’s transmission system. The main idea of the proposed algorithm is to extract the user’s hand first motor synergy’s activation level and use this trajectory as position reference to drive the slave’s actuator. Indeed, this component not only reflects the high-level intent of the operator but it also corresponds to a coordinate along the synergistic path described by the SoftHand’s joints.

To begin with, Sect. 3.1 outlines the neuroscience-based notion of hand synergies, which inspired the robotic community with a radically new control approach that aims at emulating the strategy employed by the CNS to tame the motor control complexity of the hand’s many DOFs. While a large body of research has built upon this idea, the totality of these studies—to the best of our knowledge—has considered the synergies as derived from the hand joint trajectories. The present work investigates the possibility of extending this concept toward extracting the synergistic patterns of motion of the hand from its postural descriptions in the Cartesian space of the fingertips instead of the joint space. Section 3.2 elaborates on a user study that has been conducted to investigate this possibility. On the basis of the experimental results that validated this approach, the notion of Cartesian-based synergy space is introduced. Section 3.3 describes how this paradigm is implemented into the control scheme of the proposed teleoperation framework. A characterization of the asymmetric teleoperator in then presented, where the behavior of the slave is analyzed under different inputs issued by the operator. Specifically, the response of the system to finger adduction–abduction and flexion motions is reported. The results clearly indicate the effectiveness of the approach, which enables to intuitively control the slave robotic hand from natural hand motions.

This Cartesian-based synergy port is proposed as an analogous and simpler alternative over the classical postural synergy that may suit better hand tracking systems, which do not provide direct measurement of the joint angles but are able of high resolution 6-DOF tracking of the fingertips. In such cases, the Cartesian-based synergy analog could be used for extracting from Cartesian tracking data the activation level along a synergy coordinate, alleviating the need of resorting to complex joint tracking systems or computationally expensive and potentially inaccurate inverse kinematic solutions of the hand. In addition, given a different end-effector or robotic hand with different kinematics, actuation, and posture control capabilities, this method could be tuned to extract the user’s intent along additional synergistic actions that could be then related to the commanded actions of the slave.

### 3.1 Hand Synergies, a Path toward Simplicity

The human hand is a fascinating and remarkably complex biological system in which a multitude of muscles and tendons controlling the joints’ angular excursions are simultaneously engaged to generate purposeful movements. Understanding how the brain harnesses the motor control complexity of this organ has been extensively studied, and evidences have been put forward to substantiate the idea that the Central Nervous System does not consider it as a collection of independent joints. Instead, various neuroscience analyses postulate the existence of motor primitives defining the coordinated motion of multiple joints along trajectories that are referred to as synergies (Latash, 2008). Interested readers are referred to the thorough reviews presented in Tresch et al. (2006) and Santello et al. (2013). Although the term synergy has been employed to conceptualize coordination at various levels including kinematics, i.e., joints motion coordination (Santello et al., 1998), kinetics, i.e., digit force coordination (Zatsiorsky et al., 2000), neuromuscular, i.e., multi-muscle activation patterns (Bizzi et al., 2008), or neurosensorial, as the perceptual counterpart of the motor synergies (Bicchi et al., 2011), we will use here the definition proposed in Turvey (2007), according to which a synergy is “a collection of relatively independent DOFs that behave as a single functional unit.”

This synergistic behavior is believed to result from a combination of biomechanical constraints, related in particular to the muscles and tendons spanning several joints, with neural constraints, stemming from a specific circuitry that distributes high-level commands into multiple inputs traveling along descending pathways to various muscles (Schieber and Santello, 2004). The complex interplay between these hard-wired and soft-wired constraints results in multi-DOFs coordination patterns, such that each synergy can be seen as a vector specifying the relative motion of a collection of joints, while their absolute position is controlled through modulating a single signal. Therefore, they constitute basic building modules that can be combined using a set of time-synchronized activation level trajectories that translate in principled patterns of motion and ultimately allow to produce a large repertoire of movements.

Gaining a deeper insight into the synergistic mechanism underlying the hand motor control is crucial to apprehend the high-level intentions of the user from motion analysis. Indeed, synergies are believed to be closely linked to the functional outputs of a motor behavior (d’Avella and Bizzi, 2005) and “may therefore represent the bottom of a hierarchical neural control structure in which higher neural centers operate on increasingly conceptual variables related to task-level motor performance” (Ting and McKay, 2007).

Among the many implications deriving from the synergistic organization of the hand motor apparatus, a very interesting aspect regards the dimensionality reduction of the control space it implies. Diverse techniques have been proposed to individuate the patterns of joints’ covariation that occurs when modulating the hand posture to grasp objects of diverse shapes, including Singular Value Decomposition (Mason et al., 2001) and Principal Components Analysis (PCA) (Santello et al., 1998). The latter study reveals that only two independent variables, corresponding to the levels of activation of the first two postural synergies, account for more than 80% of the variance of a dataset containing the static postures at the end of the grasp motions of a large number of imagined objects. This indicates that the hand molding motions involve principally two synergies. It is worth specifying that these observations do not imply that additional synergies represent random, task-unrelated variability. Instead, they represent effective degrees of freedom that come into play when finer posture adjustments are required, especially in order to conform to the actual geometry of the objects manipulated (Todorov and Ghahramani, 2004).

While a large amount of studies have been dedicated to the analysis of the hand postural synergies, all of them—to the best of our knowledge—have considered the synergies as extracted from the hand’s joint space description. In the present work, we propose to investigate the possibility to perform an analogous PCA-based dimension reduction analysis from the hand posture described in the fingertip Cartesian space. Note that the objective is not to discuss whether motor control strategies are encoded at the joint or task-space level by the CNS but rather to explore how they can be adequately monitored. Considering that joints and end-effector Cartesian trajectories are correlated, it is reasonable to hypothesize that the synergistic organization that has been evidentiated in the joint space would be reflected in the Cartesian space.

This approach stems from the hand exoskeleton device used at the master station, which provides a direct tracking of the fingertips 6D position trajectories. These could be used to infer the joints trajectories through implementing an inverse kinematics module. However, not only this would require the introduction of a calibration procedure that can be long and tedious to identify the user’s relevant kinematic parameters but it would also and more importantly entail a reduction of the hand posture estimation accuracy. Indeed, the intrinsic complexity of the hand anatomical organization makes accurate modeling challenging. This complexity compels to the development of simplified kinematic models so as to achieve manageable solutions and meet the computational speed requirements associated with real-time applications (Bullock et al., 2012). However, it is important to keep in mind that such simplifying assumptions (e.g., joints axis alignment, number, nature of the joints, etc.) introduce an error in the hand posture estimation. Instead, the extraction of the synergies coordinates directly from the motion data captured permits to avoid this pitfall.

In the following is presented the study that has been conducted to investigate the feasibility of identifying the hand postural synergistic paths and their respective levels of activation from the tracking of the fingertips motion in the Cartesian space.

### 3.2 Experimental Analysis of Human Grasps in Cartesian Space

An experimental study has been carried out in order to determine whether the postures adopted by the hand during grasping motions could be adequately described by a low-dimensional set of coordinates traveling along motion primitives expressed in the fingertips’ Cartesian position space. The aim was to investigate whether the preliminary results presented in Brygo et al. (2016), which considered a single subject, could be generalized to different hands kinematics.

Eight right-handed subjects—3 females and 5 males aged between 25 and 36—took part to this experiment. Ethical approval was not required for this study as per the national and institutional requirements, and written informed consent was obtained from all participants. They were instructed to wear the hand exoskeleton HEXOTRAC and to sit at a table with the upper arm resting along the body, the elbow joint right-angled, the forearm horizontal with the palm-down hand opened. They were then asked to imagine an object placed 40 cm away on the table and enjoined to shape their hand while executing a natural reach-to-grasp motion as if to pick it up, before returning to their initial configuration. They repeated this operation 5 times per object with 57 different objects, for a total number of 5 × 57 items per subject. In order to allow for comparisons, the objects used were the same as listed in Santello et al. (1998). These everyday objects require both precision and power grips and exhibit a large range of shapes in order to account for the modulation of the hand posture during natural grasping. Note that subjects were asked to imagine the objects in order to allow for the study of the motor control strategies employed by the CNS while avoiding the mechanical interference and constraints that would result from physical interactions with real objects.

The exoskeleton’s fingers’ joints position trajectories were recorded at a 1 kHz sample rate and used to compute the Cartesian position trajectory of the users’ fingertips using the equation (1) as detailed in Sect. 3.3. Finally, the data were stored in a *n_c_* × 3*m* matrix, with $n_{c}\in R$ the data collection’s number of samples and *m* = 3 the dimension of the space used to describe the position of each fingertip.

**Figure 4:**
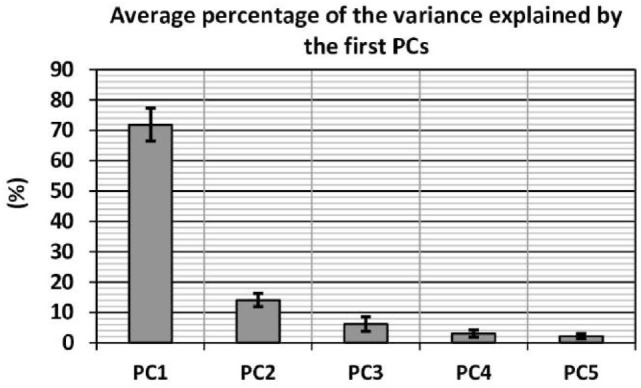
**Percentage of the variance explained by the 5 dominant PCs averaged over all subjects**. The error bars indicate the SD from the mean.

A PCA analysis has been performed to examine the internal structure of the datasets, and the percentage of the variance explained by each principal component (PC) has been computed for each subject. The averaged values over all subjects of the five largest PCs are presented in Figure 4. Additionally, the dispersion of the data from the mean is quantified for each PC by computing the associated SD, which is graphically represented on the same figure by the mean of error bars.

The first PC accounts in average for 71.8% of the variance of the data, while the first two PCs account in average for more than 85% of the variance. Furthermore, it is worth noting the low SD values, which indicate that the percentage of the variance explained by the each PC is fairly consistent among subjects. These results reveal that the 9 variables expressed in the initial base are strongly correlated. Therefore, the hand motion can be more adequately described using a new set of independent variables varying along orthogonal axis oriented in the directions maximizing the variance explained in the dataset. Through discarding the variables corresponding to the large PCs, which contribute very little to the variability of the data, it is possible to describe the hand posture during grasp motions using a low-dimensional set of variables with a minimum information loss.

This change of base can be performed using a projection matrix whose columns are the eigenvectors of the covariance matrix of the mean-centered data. In analogy to the joint space terminology, this matrix will be referred to as the Cartesian-based synergy matrix *S_x_*. Its column vectors, which are the orthogonal axes of the new base and represent the motor primitives whose linear combination describe the hand postures, will be referred to as the Cartesian-based synergies. In the rest of this work, if not stated explicitly, the term synergies will be used to refer to the Cartesian-based synergies, although the reader is invited to keep in mind that they are distinct from the joint space-based synergies as defined in the literature.

The obtained synergy matrix *S_x_*, computed once from the data collection, is then used to project in real time the user’s hand posture on the synergy space so as to extract each posture’s coordinate along the first synergy, as detailed in the following section.

### 3.3 First Synergy Position Reference Extraction

This section explains how the raw fingertips’ Cartesian position trajectories monitored by the hand exoskeleton can be mapped onto a new set of scalar variables representing the hand’s posture coordinates in the Cartesian-based synergy space. Subsequently, an experimental study is presented that analyzes the effectiveness of the proposed approach in capturing the user’s grasping intent during unconstrained motions and in translating it into adequate references to control the amount of closure of the slave hand.

The exoskeleton links’ configuration is monitored using the position encoders mounted on all joints. The 6-DOFs Cartesian trajectory of the operator’s fingertip *x*(*t*) can be computed straightforwardly using the forward kinematics of the exoskeleton fingers as shown in equation (1).

(1) $$x\left(t\right)=f_{K}\left(q\left(t\right)\right)$$

with $x\left(t\right)\in R^{6}$ the fingertip Cartesian trajectory, $q\left(t\right)\in R^{n}$ the exoskeleton finger position trajectory in joint space, *n* = 6 the number of joints per finger, and *f_K_* the exoskeleton forward kinematics function. The hand posture tracked by the exoskeleton can then be mapped from the Cartesian space to the synergy space using equation (2).

(2) $$\sigma \left(t\right)=S_{x}^{-1}X\left(t\right)$$

with *m* = 3 the number of fingers, $X\left(t\right)\in R^{3m}$ the fingertips position described in Cartesian space stacked in a single vector, $\sigma \left(t\right)\in R^{3m}$ the projection of *X*(*t*) on the synergy space, and $S_{x}\in R^{3m\times 3m}$ the hand synergy matrix. The first component *σ*_1_, which corresponds to the projection of the user’s hand posture along the first synergy, is extracted and scaled to the robotic hand’s motor range. This position reference is then tracked within inner PI controller.

**Figure 5:**
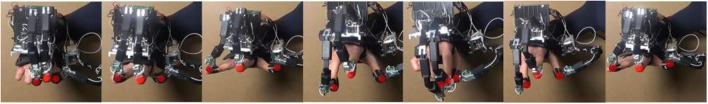
**Snapshots corresponding from left to right to the a, b, c, d, e, f, and g points in Figure 6**.

**Figure 6:**
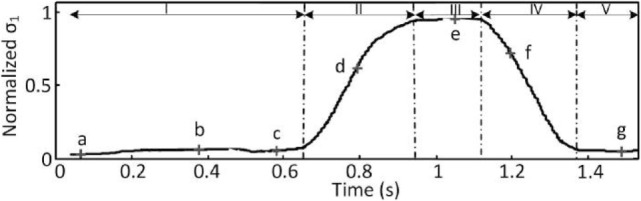
**First synergy coordinate during I: fingers abduction with opened hand, II: hand closing, III: max closure, IV: hand opening, and V: opened hand**.

An experimental study has been conducted to characterize the system’s behavior. Two types of motion were considered. During the first phase, the operator performed a fingers abduction motion in the palm plane. During the second phase, the operator was instructed to close the hand as if to grasp a sugar lump. Note that this object does not belong to the list of items used to build the synergy matrix so as to attest that this procedure extracts the grasping intent of the user independently from the exact object that is being grasped. Seven snapshots of the hand during the different phases of motion are presented on Figure 5. The extracted coordinate along the first synergy is presented on Figure 6.

Results (see also Extension 1) clearly indicate that motions outside of the first synergy do not affect the slave reference, which remains almost constant during the first phase. During the second phase, the grasping motion of the operator is properly mapped to the synergistic coordinate *σ*_1_. Therefore, the proposed teleoperation strategy allows for the intuitive control of the SH through handling natural, unconstrained motions of the operator; from the hand posture is extracted in real time the first eigengrasp coordinate that can be used to drive the SH.

While this section addressed the extraction of the grasping intent of the operator to control the closure of the SH, the following section analyzes the generation of the force feedback reference in order to render to the operator the impedance of the environment manipulated by the slave hand.

## 4 Force Feedback: Mapping a 1-DOF Grasping Torque to a 3-Finger Force Reference

In order to increase the sense of telepresence of the user, a kinesthetic feedback reflecting the interaction forces between the robotic hand and the manipulated environment is displayed by the exoskeleton.

As the SoftHand’s motor current sensing is the only available information about the amount of forces generated by the robotic hand during the manipulation, an interaction torque observer is implemented. This estimated interaction torque represents the grasping effort applied along the SH’s actuated dimension, which coincides with the hand’s first synergy. This one-dimensional reference is then mapped into a 9-D reference in the fingertips Cartesian space of the user through an inverse projection of the Cartesian-based synergy space introduced in the previous section. As the corresponding Cartesian-based synergy matrix depends on the kinematics of the hand, a novel force scaling procedure is introduced that permits to homogenize the amplitude of the force reference across users in consistency with the aim of designing a universal platform. Finally, the resulting force reference in the fingertips Cartesian space is projected on the exoskeleton’s actuated joint space in order to derive the motors torque references.

Note that another possible approach to generate force feedback references consists in mounting force/torque sensors on the fingertips of the SoftHand so as to get a direct measurement of the interaction forces. However, the choice to rely on the SoftHand’s current measurement to estimate the interaction forces stems from the overall objective of minimizing the complexity of the setup so as to achieve a highly robust and cost-effective interface. Complementarily with this goal, the present study aims at investigating the possibility to compensate for such minimal sensing system by the mean of a control layer capable of resolving the large master–slave asymmetry through mapping a scalar force reference described in the slave’s sensing space to a force reference of larger dimension described in the master’s actuated space.

After deriving the mathematical foundations underlying the mapping from the synergistic force reference to the finger-individualized force feedback in Sect. 4.1, the closed-loop performance of the bilateral system is analyzed, and a force feedback characterization is presented in Sect. 4.2.

### 4.1 Mapping a 1-DOF Grasping Torque to a 3-Finger Force Reference

Considering that the synergy matrix *S_x_* introduced in Sect. 3.2 is a linear, time-constant operator, the equation (2) can be differentiated with respect to time such that

(3) $$\dot{X}\left(t\right)=S_{x}\dot{\sigma }\left(t\right)$$

Provided that $\dot{X}$ represents a velocity, then the power in initial base can be expressed as

(4) $$F{\left(t\right)}^{T}\dot{X}\left(t\right)=P\left(t\right)$$

with $F\left(t\right)\in R^{3m}$ representing the Cartesian space force vectors applied at the fingertips stacked in a single column vector.

Introducing $z\left(t\right)\in R^{3m}$, the force vector in the synergy space, the power in this base can be defined through

(5) $$z{\left(t\right)}^{T}\dot{\sigma }\left(t\right)=P\left(t\right)$$

Therefore, applying the power balance equation between the initial and projected bases, the following force space mapping holds:

(6) $$z\left(t\right)=S_{x}^{T}F\left(t\right)$$

Considering that *S_x_* is an orthogonal matrix, the Cartesian force vector can be computed from the synergy force vector through

(7) $$F\left(t\right)=S_{x}^{-T}z\left(t\right)$$

with *z*(*t*) the force reference in synergy space defined as

(8) $$z\left(t\right)=\left[z_{1}\left(t\right)\;0\ldots 0\right]$$

such that

(9) $$z_{1}\left(t\right)=\tau_{\mathrm{int}\left(t\right)}$$

with $\tau_{\mathrm{int}}\in R$ the interaction torque applied by the slave hand on the remote environment. It is estimated from the current and position measurement at the DC motor of the SoftHand using the interaction torque observer presented in Ajoudani et al. (2014). In this module, the total torque generated by the motor is computed, and its external component is isolated from its internal component, which corresponds to the elastic torque generated by the tendons and the frictional torque arising at the joints and pulleys. The resulting interaction torque observed corresponds to the grasping effort applied by the SoftHand on its environment along the first synergy.

The Cartesian force reference is then mapped to the exoskeleton joint space using

(10) $$\tau_{\mathrm{exo}}\left(t\right)=J{\left(q\right)}^{T}F\left(t\right)$$

where $\tau_{\mathrm{exo}}\in R^{3n}$ denotes the exoskeleton torque vector, $J\left(q\right)\in R^{3m\times 3n}$ the exoskeleton Jacobian, and *n* = 6 the dimension of each finger joint space.

The force feedback reference *F*(*t*) corresponding to a given interaction torque varies across users depending on their individual synergy matrix as described by equation (7). To get comparable force feedback amplitudes across all users for the same interaction torque and to use the full torque range of the exoskeleton, user-dependent, finger-individualized force scaling gains $k_{i}\in R$ are introduced such that

(11) $$\tau_{\mathrm{exo}\;i}\left(t\right)=J_{i}{\left(q\right)}^{T}F_{i}\left(t\right)k_{i}$$

with *i* ∈ {0, 1, 2} the exoskeleton’s finger linkage considered. These gains are computed such that the maximum interaction torque $\tau_{\mathrm{int}\;\max}\in R$ that the system is expected to render is mapped to the maximum torque that the exoskeleton can deliver $\tau_{\mathrm{exo}\;\max}\in R$ when the hand of the user is closed:

(12) $$k_{i}=\frac{\tau_{\mathrm{exo}\;\max}}{J_{c\;i}{\left(q\right)}^{T}F_{i\;\max}}$$

with $J_{c\;i}\left(q\right)\in R^{m\times n}$ the Jacobian of the *i*-th linkage in closed hand configuration and $F_{i\;\max}\in R^{m}$ computed from

(13) $$F_{\max}=S_{x}^{-T}z_{\max}$$

with

(14) $$z_{\max}=\left[\tau_{\mathrm{int}\;\max}\;0\ldots 0\right]$$

**Figure 7:**
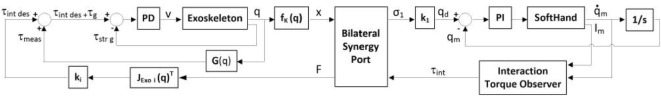
**Synergy-based bilateral teleoperation controller block diagram**.

The integration of this synergy port within its overall bilateral teleoperation control scheme as illustrated on Figure 7.

### 4.2 Closed-Loop Performances: Force Feedback Characterization

A force feedback characterization has been performed in order to analyze two main aspects. The first objective was to examine whether the direction of the forces, as computed through the proposed method and after projection on the exoskeleton’s actuated joint space, was meaningful. The second objective was to check that the amplitude of the force feedback adequately reflects the level of forces applied by the slave hand on its environment.

**Figure 8:**
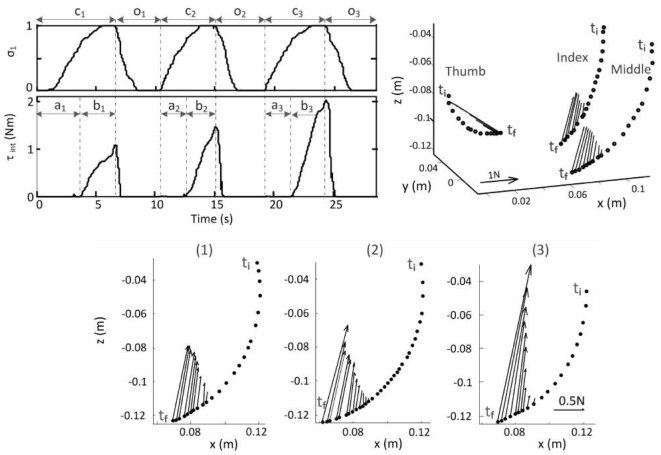
**Top left: normalized coordinate along the first synergy and interaction torque during the 3-ball grasping experiment**. *i* = 1, 2, 3: index ball grasped, c: closure hand, o: opening hand, a: object’s enveloping phase, b: object’s squeezing phase. Top right: tracking of the fingertips and forces applied during the first ball grasping between *t_i_* (initial time) and *t_f_* (final time). Bottom: position trajectory of the middle fingertip and forces applied during the grasping of the balls (1), (2), and (3) between *t_i_* (initial time) and *t_f_* (final time).

To this end, an experiment has been conducted during which a user was instructed to control the closure of the SoftHand so as to grasp and squeeze three foam balls (1), (2), and (3) of increasing stiffnesses. The fingertips position trajectories were monitored by the exoskeleton and projected on the synergy space so as to derive the coordinate along the first synergy *σ*_1_(*t*) used to control the SH. Results are presented in Figure 8. The interaction torque τ*_int_*(*t*) developed during each ball squeezing was estimated by the torque observer. Both *σ*_1_(*t*) and τ*_int_*(*t*) are shown on the top left plot. The direction of the forces actually applied at the fingertips by the exoskeleton is shown on the top right plot. To facilitate the comparison of the force amplitudes in the three conditions, the bottom plot presents a projection in the finger’s plane of motion of the forces applied on the middle finger during the squeezing of the each ball.

These plots indicate that forces are applied along directions that oppose the user’s hand closure along the first synergy, making coherent the synergy-based haptic closed-loop approach. Furthermore, the amplitude of the force applied at each fingertip increases with the grasping force, properly reflecting the interaction at the slave side. This behavior is also shown on Extension 1. After experimentally validating the effectiveness of the force feedback policy proposed, the possibility of introducing a simplified calibration procedure has been analyzed as discussed in the following section.

## 5 A Simplified Calibration Procedure

In the approach presented previously, the Cartesian-based synergy matrix needs to be computed for each user since it depends on the hand’s kinematics and in particular on the phalanges’ length. However, the associated procedure described in Sect. 3.2 can appear a bit long and wearing. As such, it might hinder the quick and easy use of the system by any new user. Hence, the idea to evaluate the possibility to project the fingertip position trajectory on a unique, user-independent synergy matrix (which will be referred to as the Simplified Calibration Procedure or SCP) rather than on the user’s own synergy matrix (which will be referred to as the Full Calibration Procedure or FCP). In order to analyze the feasibility of this approach, the difference stemming from the use of the full versus simplified calibration procedure is examined both in terms of position control of the slave’s hand in Sect. 5.1 and in terms of force feedback in Sect. 5.2.

### 5.1 Position Control: A Comparative Analysis

To analyze the effect of relying on the simplified rather than full calibration procedure, the fingertips position trajectory $X_{\mathrm{user}}\left(t\right)\in R^{3m}$ of 7 users were recorded using the exoskeleton during a hand closing-opening motion.^2^

These trajectories have then been projected on each user’s own synergy matrix [see equation (15)] and on another unique synergy matrix *S_x ref_*^3^ [see equation (16)].

(15) $$\begin{array}{l} \sigma_{1}\left(t\right)=S_{x\;\mathrm{user}}^{-1}\;X_{\mathrm{user}}\left(t\right) \end{array}$$

(16) $$\begin{array}{l} \sigma_{1}^{\prime }\left(t\right)=S_{x\;\mathrm{ref}}^{-1}\;X_{\mathrm{user}}\left(t\right) \end{array}$$

**Figure 9:**
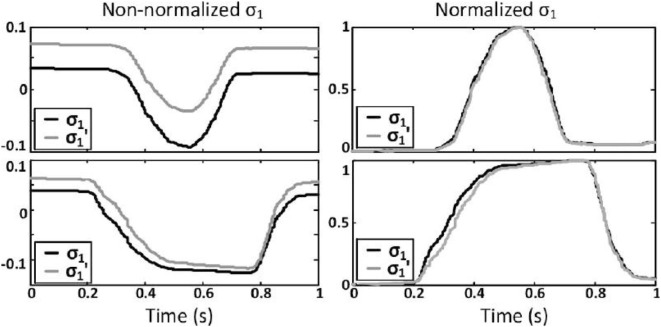
**Comparison of ***σ*_1_** and $\sigma_{1}^{\prime }$ before (left) and after (right) normalization**. The first row corresponds to a typical case (NRMSE = 1.85%), while the second row represents the subject with the largest NRMSE, or worst case (NRMSE = 4.25%).

Results indicate that the coordinate along the first synergy obtained through the SCP $\sigma_{1}^{\prime }\left(t\right)$ differs from the one obtained through the FCP *σ*_1_(*t*), as shown for two of the subjects on Figure 9 (left hand side).

To reduce the intersubject variability as well as to scale the position reference to the slave’s range of motion, the raw coordinate along the first synergy is subsequently normalized. This procedure simply requires to record the fingertips’ position of each user with the hand fully opened and fully closed so as to compute the associated coordinates $\left\{\sigma_{1\;\min},\sigma_{1\;\max}\right\}.$ These values are used to scale in real time the coordinate along the first synergy, reflecting the user’s current degree of hand closure, to a value between 0 and 1. The normalized coordinates obtained from the two calibration procedures are then compared. They present a high degree of similarity, as shown for two of the subjects on Figure 9 (right hand side). To quantify the error introduced by the simplified calibration procedure with respect to the full one, the average Normalized Root-Mean-Square Error (NRMSE) between *σ*_1_(*t*) and $\sigma_{1}^{\prime }\left(t\right)$ after normalization is computed for each subject and presented in Table 1.

**Table 1:** **NRMSE between *σ*_1_(t) and $\sigma_{1}^{\prime }\left(t\right)$ after normalization for each subject**.

Subject id	S1	S2	S3	S4	S5	S6	S7
NRMSE (%)	1.05	4.25	1.9	1.81	3.88	1.52	1.85

With an averaged NRMSE across all subjects of 2.32%, $\sigma_{1}^{\prime }\left(t\right)$ and *σ*_1_(*t*) are deemed to be similar enough, after normalization, to consider that the SCP does not affect the user’s ability to intuitively and precisely control the position of the slave’s hand.

In the following sections, the effect of using the simplified rather than the full calibration procedure are examined from the point of view of the force feedback generated.

### 5.2 Force Feedback: A Comparative Analysis

This section addresses the comparison of the force feedback reference generated through the FCP [*F*(*t*); see equation (17)] in the one hand, and through the SCP [$F^{\prime }\left(t\right)$; see equation (18)] in the other hand.

(17) $$\begin{array}{l} F\left(t\right)=S_{x\;\mathrm{user}}^{-T}\;k\;z\left(t\right) \end{array}$$

(18) $$\begin{array}{l} F^{\prime }\left(t\right)=S_{x\;\mathrm{ref}}^{-T}\;k\;z\left(t\right) \end{array}$$

**Figure 10:**
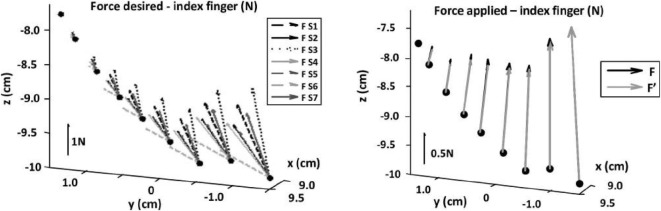
**Left: comparison of the force feedback desired across subjects for the index finger**. Right: comparison of the force feedback computed through the simplified vs full calibration procedure.^4^

To this end, an operator wearing the hand exoskeleton remotely controlled the squeezing of a ball by the slave hand. The interaction torque was recorded, and the corresponding force feedback references were computed from equation (17) using the 7 subjects’ synergy matrices, as determined in Sect. 3.2, and are presented in Figure 10 (left hand side).

Results are clearly different across subjects, indicating that the user’s synergy matrix does affect the desired force feedback. Therefore, if a master device capable to apply such forces is to be used, relying on the simplified calibration procedure would alter the kinesthetic feedback in a non-negligible way. It would be interesting to analyze whether users feel more comfortable when the force feedback reference is generated using their own synergy matrix rather than a user-independent synergy matrix, i.e., if it does alter the teleoperation performances.

That being said, it is worth noting that the differences across subjects’ mainly regard the force direction. However, the feedback device used in the proposed implementation is highly underactuated, such that the direction of the force that can be applied is posture-dependent. Consequently, in order to analyze the influence of the calibration procedure on the force feedback that is actually displayed to the user, the desired forces *F*(*t*) and $F^{\prime }\;\left(t\right)$ need to be compared after projection on the exoskeleton’s actuated joint space using equation (10). These forces are computed for all subjects and presented for one of them on Figure 10 (right hand side).

After projection on the exoskeleton’s actuated joint space, the desired forces synthesized from the two calibration procedures are parallel, such that they can be compared through quantifying their norm difference using equation (19).

(19) $$\mathrm{error}\;\mathrm{norm}\;\mathrm{force}=\left|\frac{∥F∥-∥F^{\prime }∥}{∥F^{\prime }∥}\right|$$

This error has been computed for the 7 subjects, and the averaged values for all data points and all fingers are presented in Table 2.

**Table 2:** **Force norm error for each subject**.

Subject id	S1	S2	S3	S4	S5	S6	S7
Error norm force (%)	2.02	3.28	5.71	3.79	4.13	2.88	11.31

With an average value of the force norm error across all subjects of 4.73%, the influence of the calibration procedure on the force feedback applied by the hand exoskeleton is deemed marginal.

Finally, the analysis presented in this section indicates that the bilateral synergy-based approach proposed is robust enough to handle an approximation on the synergy matrix used. As such, a unique, user-independent synergy matrix can be used for users with different hand kinematics. Such simplified calibration procedure is considered to be adequate to generate both the position reference for the slave hand and the force feedback references for the hand exoskeleton device. In order to validate the hypothesis that users are able to effectively use the resulting interface, an experimental study has been conduced and is presented in the following section.

## 6 Experimental Assessment of the Framework Performances

In order to convert the tele-grasping interface into a tele-manipulation one, the proposed framework is further enriched with a robotic arm at the slave side, a tracking system at the master side as well as an associated control system, as described in Sect. 6.1. The effectiveness of the overall framework in enabling an operator to reach, grasp, and transport remote objects is then experimentally evaluated during two experiments presented in Sect. 6.2 and 6.3.

### 6.1 Robotic Arm Teleoperation

The synergy port developed permits the bilateral teleoperation of the SoftHand from monitoring the user’s finger posture using a hand exoskeleton. However, a grasp is completely defined by the combination of the hand’s intrinsic and extrinsic DOFs, as underlined in Ciocarlie and Allen (2009). Indeed, the stable grasp of an object located in its environment requires the control of the wrist position and orientation during the reaching phase so as to place the robotic hand in an adequate configuration with respect to the object such that the further closure of the hand during the pre-shaping and grasping stages leads to a force closure grasp.

**Figure 11:**
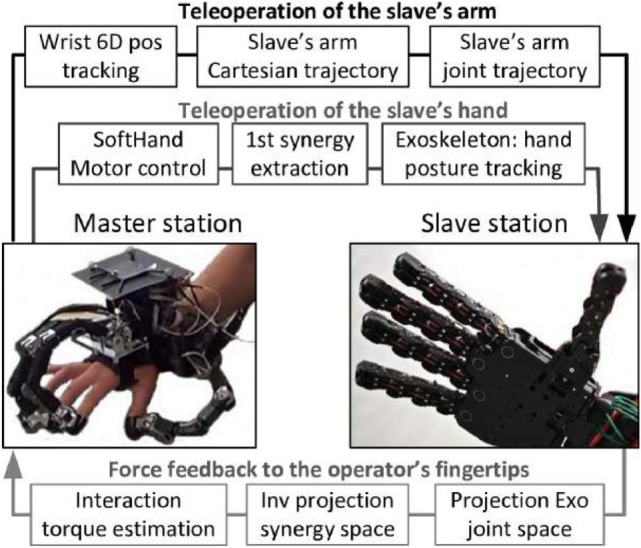
**Control principle of the tele-manipulation framework implemented**.

To address this requirement and enable the operator to manipulate remote objects, the teleoperation framework is further enriched with a robotic arm on which the SoftHand is mounted. Additionally, the vision-based motion capture system Optitrack is integrated at the master station, and a visual tracker is attached to the base of the exoskeleton. This augmented interface enables to monitor the position and orientation of the operator’s wrist in the world frame. This signal is processed (filtering, frame transformations) and used as position reference trajectory in Cartesian space for the robotic arm’s wrist. The control principle of the overall framework is schematized on Figure 11. Two robotic arms have been integrated to the framework: the humanoid robot COMAN in the one hand, and on the other, the KUKA LWR manipulator. While the Cartesian controller of KUKA could be used straightforwardly, with COMAN a second controller layer has been implemented, as described in Brygo et al. (2014), to derive through inverse kinematics the arm’s joints position trajectory from the robot’s wrist 6D position trajectory reference.

**Figure 12:**
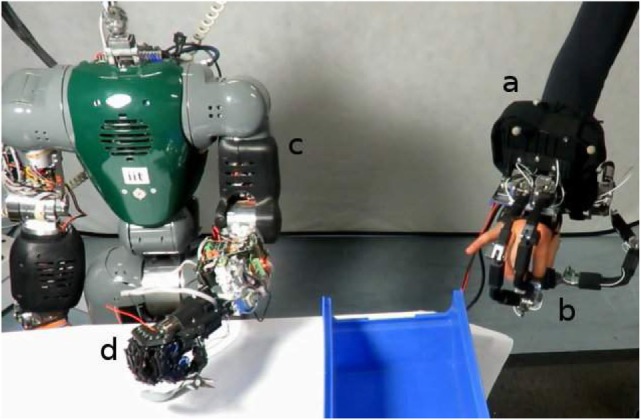
**An augmented framework for performing remote tele-manipulations using the humanoid robot COMAN**.

**Figure 13:**
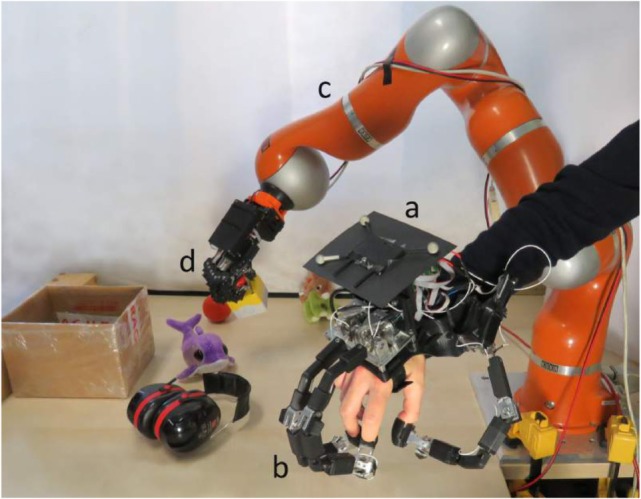
**An augmented framework for performing remote tele-manipulations using the KUKA LRW robot**.

So finally, the setup consists of a visual tracker (a) attached to the hand exoskeleton (b), a robotic arm (c) on which is mounted the SoftHand (d) as illustrated on Figures 12 and 13.

The effectiveness of the overall tele-manipulation framework is then experimentally evaluated, as presented in the following two sections as well as in Extension 1.

### 6.2 Tele-Manipulation with the Humanoid Robot COMAN

A first experiment has been conducted that focused on evaluating the versatility of the platform through assessing the possibility to execute the stable grasp of a large range of different objects. To this effect, the user was instructed to drive the arm of the humanoid robot COMAN fitted with the SoftHand so as to reach for one of the items placed on a table, securely grasp it, lift it, and drop it off on a case, as shown in Figure 12.^5^ The operator was invited to repeat this reach–grasp–transport–release cycle with a collection of 11 objects counting a remote control, a screwdriver, a water bottle, a phone charger, a pair of pliers, an emergency button, a chewing-gum box, a noise-canceling headset, as well as a dinosaur, a whale, and a leopard cuddly toys. These articles have been selected upon the diversity of their volumes, their geometries, and the mechanical properties of their materials that reflect the heterogeneity of the objects typically populating human workspaces.

After a short trial period to get used to the setup and the geometry of the objects, the operator successfully completed the task, grasping in a stable way and transporting all the items without dropping them. Besides illustrating the effectiveness of the proposed human–machine interface, this experiment demonstrates that the proposed platform achieves the level of versatility required to manipulate objects exhibiting very different shapes and impedances. Such endowment confers to the framework a large flexibility, which makes it suitable for a variety of manipulation scenarios in unconstrained environments.

Additional characteristics of interests are examined in a second experiment, as presented in the following section.

### 6.3 Tele-Manipulation with the KUKA LWR Manipulator

To further evaluate the effectiveness of the proposed bilateral tele-manipulation interface, a second experiment has been carried out. With the twofold purpose of increasing the reachable workspace of the slave and of showcasing how the modularity of the framework permits the straightforward interchange of manipulators, the KUKA LWR manipulator has been used as robotic arm.

This experiment focused on assessing the universality of the proposed interface. The term universality refers here to a dual concept, such that the first objective was to analyze the capacity of the framework to accommodate for different users with different hand sizes and to rely on the simplified calibration procedure described in Sect. 5. The second aim was to evaluate the level of intuitiveness of the overall platform.

To this end, 10 subjects aged from 24 to 34 years took part to the experiment. Ethical approval was not required for this study as per the national and institutional requirements, and written informed consent was obtained from all participants. Their index and middle fingers’ lengths, respectively, ranged between 8.3–9.9 and 9.3–11 cm. None of them ever used the platform before, and 5 of them never teleoperated a robot before. They were given 5 min to get used to the setup before starting the task, which consisted in picking up one by one the 6 items (a whale and a dinosaur soft toys, a phone charger, a noise-canceling headset, a remote control, and an emergency button) lying on the table and place them in a 21 cm × 18 cm × 15 cm box, labeled with the letter (e) on Figure 13. At the end of the experiment, subjects were invited to fill a form rating their experience on a 1–5 scale.

**Figure 14:**
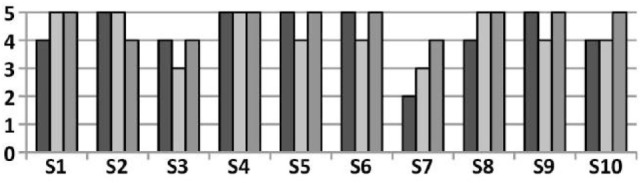
**Rating of the tele-manipulation platform by each subject *S_i_*—from the darker to the paler: level of intuitiveness, level of overall appreciation, level of confidence**.

Results indicate that all the subjects successfully completed the task and managed to place all the objects in the target box in an average time of 3.4 min. To quantify the perceived intuitiveness of the overall platform, subjects were asked to answer to the question “How easy was it to control the slave robot (KUKA + SoftHand)?” on a scale from 0 (very difficult, non-intuitive) to 5 (very easy, very intuitive), which was rated as 4.3/5 in average. The average rating to the question “In order to understand how confident you are in using this platform, rate how stable/predictable you perceived it,” was 4.2/5. Finally, subjects were asked to indicate whether they liked using the interface in overall terms, from 1 (“No, I got frustrated. The slave was not doing what I wanted, or it was too difficult to achieve.”) to 5 (“Yes, it is very nice to use”), which received a 4.7 rating in average. The distribution of the ratings for all three questions is shown on Figure 14.

Finally, this experiment clearly indicates that (i) the simplified calibration procedure is suitable and provides the bilateral control framework with the robustness and flexibility required to accommodate for different users with different hand sizes, relying on a simple 30 s calibration step; (ii) the platform is highly intuitive and allows naive users with no prior training to perform remote manipulation of objects of diverse shapes and impedances; (iii) the interface is perceived as likable and reliable, enabling to execute the desired operations.

## 7 Conclusion

This work presents a novel bilateral control framework allowing for intuitive tele-manipulations of objects of various shapes and impedances using highly asymmetric master–slave systems. The keystone of the proposed control architecture consists of a bilateral Cartesian-based synergy port, which is introduced as a tool mapping the user’s fingertips Cartesian space to the directions oriented along the grasp principal components. This module builds upon the results of an experimental study that revealed the possibility to describe the postures adopted by the human hand during grasping motions using a low-dimensional set of coordinates traveling along motion primitives expressed in the fingertips’ Cartesian position space. A 1-min calibration procedure has been developed that enables the platform to accommodate different operators’ hand kinematics. Using this module, both the slave’s motion and the master’s force feedback reference trajectories are designed in the Cartesian synergy space.

As such, the proposed control framework addresses two main challenges associated with the design of tele-manipulation interfaces. First, it effectively handles master–slave systems exhibiting large asymmetries at the kinematic, sensing, and actuation levels. This permits, through accepting natural manipulative motions as input, to abstract the operator from the setup’s complexity so as to achieve a highly intuitive interface. Second, the bilateral control policies are synthesized in the hardware-independent hand synergy space. This makes possible the implementation of a 2-layer architecture where the high-level manipulation strategies can be designed in abstraction from the devices’ specifics that are encapsulated in the low-level layer. Such modularity not only enables to flexibly interchange end-effectors according to each task but it also promotes the synthesis of universal manipulation algorithms. As such, the present work aims at fostering the development of unified tele-manipulation control frameworks and proposes the synergies as the alphabet of a common language for robotic hands control.

In order to evaluate the effectiveness of the proposed approach, the synergy port has been implemented into the control system of a highly asymmetric tele-manipulation platform. The underactuated 3-finger hand exoskeleton HEXOTRAC permits to monitor the user’s hand posture and to display a kinesthetic feedback of the interaction forces, while the synergy-driven robotic hand SoftHand is used as slave end-effector to manipulate the remote environment. In addition, the position and orientation of the user’s wrist is tracked by the vision-based motion capture system Optitrack and used as reference trajectory to drive the robotic arm on which the SoftHand is mounted.

Experiments have been performed with the humanoid robot COMAN as well as with the KUKA LWR manipulator. Results indicate that (i) the control framework adequately extracts the grasping intent of the user during natural motions, maps this input into references for the slave hand to perform the desired manipulative task, and reflects the impedance of the manipulated environment to the user; (ii) the platform is highly intuitive and allows users with no prior experience to securely grasp and transport objects exhibiting a large range of shapes and impedances after only a few minutes of familiarization with the system; (iii) not only do the hardware and the control solutions permit to accommodate users with different hand kinematics but they also allow for a fast donning and a short calibration procedure, turning the platform into a universal and fully practical interface.

Finally, this work investigated the possibility to use largely underactuated master and slave devices fitted with minimal sensing systems so as to achieve a simple and highly robust tele-manipulation interface and analyzed the possibility to compensate for the associated hardware limitations by the mean of an adequate control strategy. Results indicate that the proposed control framework elegantly resolves the master–slave asymmetries and provides a universal and flexible interface for performing intuitive and effective tele-manipulations.

## Author Contributions

AB: theoretical contribution: equations formulation of the synergy port, development of the calibration procedure, implementation of the software architecture (control framework, interface with all robotic systems, and experimental tools); experiments: design, realization, and analysis; video; and paper writing. IS: mechanical design of the hand exoskeleton; paper revision. GG: idea of the force feedback synthesis through inverse projection on the synergy space. NT: idea of the Cartesian-based synergy mapping to control the SH.

## Conflict of Interest Statement

The authors declare that the research was conducted in the absence of any commercial or financial relationships that could be construed as a potential conflict of interest.

## Supplementary Material

Video S1 **Synergy-based bilateral port: a universal control module for intuitive tele-manipulations using asymmetric master-slave systems**.

^1^central nervous system.

^2^These subjects belong to a panel of 5 men and 2 women aged from 25 to 36 years.

^3^*S_x ref_* is the synergy matrix of an 8*th* user (female, 9-cm index finger).

^4^[Right hand side figure] This worst-case plot shows the forces computed for the subject with the largest error as defined in equation (19) for the index finger (*error norm force* = 6.92%).

^5^Note that the right SH is mounted on the left arm because COMAN’s right arm was under maintenance at the time of this experiment.
